# Effects of Carbonated and Electrolyte-Added Water on Body Fluid Regulation and Ingestive Behavior During Ad Libitum Rehydration Following Mild Hypohydration

**DOI:** 10.3390/nu18121846

**Published:** 2026-06-08

**Authors:** Akira Takamata, Natsumi Kosugi, Nanako Sakagawa, Aoi Takahashi, Fuka Nishino, Mio Nishimaki, Yasushi Tanaka, Makoto Kobayashi, Chihiro Nitta, Moeno Aihara, Yuka Hasegawa, Motoko Kobayashi, Takanobu Takihara

**Affiliations:** 1 Department of Environmental Health, Nara Women’s University, Kitauoya Nishimachi, Nara 630-8506, Japan; yan_kosugi@cc.nara-wu.ac.jp (N.K.); skgwnnk34@gmail.com (N.S.); vc.taoi0122@gmail.com (A.T.); waf_nishino@cc.nara-wu.ac.jp (F.N.); mnishima@mail.doshisha.ac.jp (M.N.); 2 Central Research Institute, ITO EN, Ltd., Makinohara 421-0516, Japan; ya-tanaka@itoen.co.jp (Y.T.); m-kobayasi@itoen.co.jp (M.K.); c-nitta@itoen.co.jp (C.N.); m-aihara@itoen.co.jp (M.A.); yuk-hasegawa@itoen.co.jp (Y.H.); t-takihara@itoen.co.jp (T.T.); 3 Marketing Department, ITO EN, Ltd., Tokyo 151-8550, Japan; mo-kobayashi@itoen.co.jp

**Keywords:** voluntary drinking, diuresis, fluid homeostasis, fluid retention, spontaneous fluid intake, stomach discomfort, taste, thirst, water-electrolyte balance

## Abstract

**Purpose**: Maintaining fluid homeostasis is crucial, and replenishing electrolytes—particularly sodium (Na^+^)—is critical for effective rehydration after thermal hypohydration. Despite the growing consumption of carbonated water (CW), its impact on post-hypohydration body fluid balance, either alone or combined with electrolytes, remains inadequately investigated. Therefore, this study was designed to examine the separate and interactive effects of carbonation and electrolyte provision in rehydration fluids—utilizing a 2 × 2 factorial design (carbonation × electrolytes)—on ingestive behavior and systemic fluid balance following mild thermal stress-induced hypohydration. **Methods**: Subjects (eight women and seven men, age range: 19–25 years) were dehydrated by performing three bouts of stepping exercise for 20 min, separated by 10 min of rest, at 25 °C. Following the dehydration protocol, subjects ingested pure water (W), CW (gas volume, 3.43 ± 0.20 (mean ± standard deviation)), electrolyte-added water (EW), or electrolyte-added CW (ECW) (gas volume, 3.04 ± 0.29 (mean ± standard deviation)) ad libitum. EW and ECW contained 19 mEq/L Na^+^. We assessed ingestive behavior and body fluid balance during the 180 min rehydration period. **Results**: The dehydration protocol induced hypohydration by ~10 g/kg body weight (~1% of body weight). Cumulative fluid intake was greater in the W trial than in the CW, EW, and ECW trials. Cumulative urine output was greater in the W and CW trials than in the EW trial. The fluid retention ratio was greater in the EW and ECW trials than in the CW trial. Consequently, the final fluid recovery was lower in the CW trial compared to the W, EW, and ECW trials; however, the combination of carbonation and electrolytes (ECW) did not significantly surpass the non-carbonated trials (W and EW) due to the reduced intake volume caused by carbonation. **Conclusions**: The data suggest that ad libitum ingestion of carbonated water is less effective than plain water for recovering from mild hypohydration (~1% body weight loss), as it reduces spontaneous fluid intake. However, electrolyte supplementation mitigates this reduced recovery by attenuating diuresis and consequently improving fluid retention ratio.

## 1. Introduction

Maintaining fluid homeostasis is crucial to sustaining physiological function and, consequently, overall health [1,2]. It is well established that hypohydration increases the risk of heat-related illness by impairing thermoregulatory responses to heat stress, such as sweating and cutaneous vasodilation [3,4,5,6]. Furthermore, maintaining optimal hydration status throughout life may contribute to a decreased prevalence of degenerative diseases, including cardiovascular and metabolic diseases [2].

Recently, consumption of unsweetened carbonated water (CW), or sparkling water, has been increasing [7]. This trend is primarily attributed to increasing health consciousness and the associated effort to avoid sugar and artificial sweeteners [7,8,9,10]. Because CW is generally perceived as highly palatable while being calorie-free, it has become a popular choice for consumers concerned about obesity and diabetes [8]. Furthermore, some studies suggest that in resting humans exposed to heat stress [11] and post-exercise in heat [12], CW ingestion, compared to plain water ingestion, increases mean arterial pressure, cerebral blood flow, and oral and whole-body exhilaration while decreasing sleepiness. These physiological and sensory characteristics could potentially influence the effectiveness of rehydration by modifying fluid intake via fluid palatability, by modifying information from the oral and gastrointestinal tract, and/or by modifying renal responses.

However, the effect of ad libitum ingestion of unsweetened CW on body fluid balance after mild hypohydration has not been fully elucidated. Maughan et al. [13] reported that, following ingestion of one liter of sparkling water, urine output for 4 h was not different from the response to the same volume of plain water ingestion in euhydrated individuals. Furthermore, previous research evaluated the effectiveness of carbonation only when the fluid volume was strictly prescribed and controlled [14]. Critically, however, such forced-drinking protocols do not reflect ingestive behavior in everyday life, where fluid intake is driven by voluntary drinking behavior. Therefore, investigating ad libitum intake is essential to mimic real-world rehydration scenarios and clarify how carbonation actually influences voluntary drinking behavior and subsequent fluid balance.

Electrolyte supplementation, especially sodium (Na^+^), is essential for recovery from thermally induced hypohydration, as thermal dehydration causes deficits in both water and electrolytes [15,16]. Most previous findings regarding the effectiveness of electrolyte supplementation were obtained from uncarbonated fluids, and a different taste perception between electrolyte-added plain and carbonated water would affect ingestive behavior and subsequent body fluid balance. Nevertheless, the effect of electrolyte supplementation to CW on ingestive behavior and subsequent body fluid balance after hypohydration remains unknown.

The present study therefore aimed to examine both the separate and interactive effects of carbonation and electrolyte provision in rehydration fluids on ingestive behavior and systemic fluid balance subsequent to mild hypohydration induced by thermal stress. To better understand how carbonation and electrolyte supplementation influence fluid intake and recovery from mild thermally induced hypohydration, subjects were given free access to four distinct rehydration fluids—plain water (W), CW, electrolyte-added water (EW), and electrolyte-added carbonated water (ECW)—and their fluid balance was examined by measuring fluid intake and urinary output. We hypothesized that following mild thermal hypohydration (~1% of body weight), electrolyte supplementation to CW (ECW) would enhance the fluid retention ratio and thereby improve body fluid recovery at 180 min compared to CW. Additionally, we investigated how carbonation interacts with electrolyte provision to affect variables such as voluntary fluid intake and urine output.

## 2. Materials and Methods

All experimental protocols and procedures in the present study were performed in accordance with the ethical standards laid down in the 1964 Declaration of Helsinki and its later amendments and were approved by the Review Board on Human Experiments of Nara Women’s University (Approval #23-47). Subjects were recruited via flyers at Nara Women’s University and nearby universities. After the experimental protocol and procedures, including benefits, risks, and compensation, were fully explained, subjects provided their written informed consent prior to participating in the present study. We investigated the separate and combined effects of carbonation and electrolyte supplementation in rehydration fluid on ingestive behavior and body fluid balance following thermally induced mild hypohydration. This study is registered with the UMIN Clinical Trials Registry (UMIN-CTR; Registered # UMIN000054493).

### 2.1. Experimental Design

This study employed a randomized, repeated-measures, crossover, single-blind design to evaluate the separate and interactive effects of beverage carbonation and electrolyte supplementation on body fluid balance. All subjects participated in four experimental trials—pure water (W), carbonated water (CW), electrolyte-added water (EW), and electrolyte-added carbonated water (ECW)—with each trial separated by a washout period of more than 3 days. The order of the beverage conditions was randomly assigned and counterbalanced using an online randomization tool (www.randomizer.org; accessed on 15 June 2024). To ensure single-blinding, the rehydration fluids were prepared by an independent researcher and served in identical, semi-transparent pet bottles with a screw cap; the participants were blinded to whether the beverages contained electrolytes, though complete blinding to carbonation was inherently impossible due to the nature of the fluid. Allocation concealment was maintained until all experimental trials and primary data collections were completed.

### 2.2. Subjects

Sixteen subjects (eight women and eight men) were enrolled; however, one male participant withdrew from the study. Data are presented for the remaining 15 subjects (8 women, 7 men). All subjects were free from medication. The anthropometric characteristics of the subjects are shown in Table 1.

For the female subjects, experiments were scheduled to ensure a similar ovarian phase within each individual (four participants were tested in their follicular phase, and the other four in their luteal phase). The primary rationale for this balanced inclusion was to minimize any potential confounding biological variation and ensure an even counterbalancing across the four crossover trials, rather than to perform an independent statistical comparison between the phases, given that the ovarian phase reportedly does not affect body fluid balance regulation during hypohydration induced by 24 h fluid restriction [17] or by acute fluid replacement after thermal dehydration [18].

To examine the effectiveness of rehydration fluids among the four experimental conditions, the required sample size was determined via a power analysis using G*Power (version 3.1). Based on an estimated mean difference in fluid retention ratio of 15% with a standard deviation (SD) of 20% (yielding a Cohen’s *f* of 0.375), we calculated that 13 subjects would be required to achieve a statistical power of 80% (1 − β = 0.80) at a significance level of α = 0.05. Considering the potential for participant attrition across the four trials, a total of 16 subjects were initially enrolled in the study. This estimation was based on a previous study reporting that body fluid retention under dehydration by ingesting plain water was ~50% with an SD of 20% [16,19]. Because electrolyte (sodium) supplementation strongly increases fluid retention by at least 15–20% compared to plain water [16], assuming a relatively large effect size (Cohen’s *f* = 0.375) was physiologically justified and appropriate for capturing the clear, primary impact of electrolyte provision on fluid balance variables. Data from 15 subjects are presented in this study.

### 2.3. Rehydration Fluids

To test the effect of ingesting fluids with carbonation and electrolyte supplementation on body fluid regulation after mild hypohydration, we tested four rehydration fluids: pure water (W), carbonated water (CW; gas volume 3.43 ± 0.20, mean ± standard deviation (SD)), electrolyte-added water (EW), and electrolyte-added carbonated water (ECW; gas volume 3.04 ± 0.29, mean ± SD). Both electrolyte-added solutions (EW and ECW) contained 19 mEq/L of sodium (Na^+^). This specific concentration was selected to mimic the electrolyte profile of commercially available, widely consumed sports drinks in Japan (typically ranging between 10 and 20 mEq/L), ensuring the practical relevance of the fluid to everyday rehydration scenarios while maintaining optimal fluid palatability. All rehydration fluids were freshly prepared in a semi-transparent pet bottle with a screw cap, refrigerated before use, and utilized within one week of production. The compositions of the rehydration fluids are shown in Table 2.

### 2.4. Protocol

Subjects refrained from heavy exercise for 24 h and from alcohol, caffeinated beverages, and salty food for 16 h before the experiment, which was carefully verified via verbal confirmation upon arrival at the laboratory. They had a light breakfast (400 kcal; Calorie Mate, Otsuka Pharmaceutical Co., Tokyo, Japan) and a bottle of water (560 mL) before reporting to the laboratory. The subjects voided, entered the room, and kept a seated position at an ambient temperature of 25 ± 1 °C for 60 min as a control period. At the end of the control period, baseline measurements of tympanic temperature and heart rate (HR) were performed. Subjective thirst, mouth dryness, refreshment, sleepiness, thermal comfort, and stomach discomfort were also assessed by visual analog scale (VAS). Then, urine collection and undressed body weight measurements were performed. After the control period, the subjects performed 3 bouts of intermittent step-up and -down exercise for 20 min, separated by a 10 min resting period, at 25 ± 1 °C. The height of the step was 25 cm for subjects > 160 cm and 20 cm for subjects ≤ 160 cm. The stepping rate was approximately 80 steps/min (or about 20 up and down cycles per minute); however, small differences in the rate (less than 5 steps/min) were allowed, as the purpose of this protocol was to induce hypohydration through perspiration. Heart rate, monitored continuously during the exercise bouts, typically ranged between 130 and 150 beats/min during the exercise, with no noticeable differences observed within individual subjects among the four trials.

After the dehydration protocol, subjects sat for 30 min at a room temperature of 25 ± 1 °C to equilibrate body fluid distribution. At the end of the equilibrium period, measurements were conducted for tympanic temperature and HR. Subjective sensations were also assessed using the VAS, and urine sampling and undressed body weight measurements were performed.

Following these measurements, subjects were instructed to ingest W, CW, EW, or ECW at 10 °C ad libitum over the 180 min rehydration period. Subjects retrieved and consumed the solutions from a portable refrigerator whenever they desired, with the temperature in the refrigerator maintained at 10 °C. For each trial, five 500 mL semi-transparent PET bottles (totaling 2.5 L of fluid) were prepared and placed inside the refrigerator. Subjects drank freely from the first bottle; once a bottle was completely emptied, they proceeded to open the next fresh bottle to continue ingestion. Subjects were explicitly instructed that additional bottles would be provided upon request if they wished to drink more than the initial 2.5 L; however, no participant requested any additional fluid. To minimize carbonation loss, the bottles were kept tightly capped at all times except during the brief moments of fluid consumption, with subjects instructed to open the cap only when drinking and to close it immediately afterward. They remained seated during the rehydration period except for during urine collection and body weight measurement. After the first bout of fluid ingestion, subjects evaluated the perceived taste of ingested fluid using VAS. At 0.5 h, 1 h, 2 h, and 3 h after the onset of the rehydration period, tympanic temperature and HR measurements were conducted. Subjective sensations were also assessed using VAS, and urine sampling and undressed body weight measurements were performed at each time point.

### 2.5. Body Fluid and Physiological Measurements and Calculations

Body fluid balance was determined from the undressed body weight change measured with an electric balance. Specifically, total body fluid balance (g/kg body weight) at each time point during the rehydration period was calculated as follows:

$$Fluid\;balance\;(g/kg)=\frac{BW\left(t\right)(g)-BW_{baseline}\;(g)}{BW_{baseline}\;(g)}\times 1000$$

where BW(t) is the undressed weight at a given time point (g) and BW_baseline_ is the baseline body weight measured at the end of the control period (g). The body fluid recovery ratio (%) and the net fluid retention ratio (%) at 180 min after the onset of rehydration were defined using the following formula:

$$Fluid\;recovery\;ratio\;(\%)=\frac{\mathrm{Fluid}\;\mathrm{Gain}\;\left(g\right)}{\mathrm{Total}\;\mathrm{Fluid}\;\mathrm{Loss}\;\mathrm{during}\;\mathrm{Dehydration}\;\left(g\right)}\times 100$$

where Fluid Gain is net fluid gain calculated by subtracting urine output from ingested fluid. Additionally, the fluid retention ratio at 180 min was defined as follows.

$$Fluid\;retention\;ratio\;(\%)=\frac{\mathrm{Cum}.\;\mathrm{Fluid}\;\mathrm{Intake}\;\left(g/\mathrm{kg}\right)-\mathrm{Cum}.\;\mathrm{Urine}\;\mathrm{Output}\;\left(g/\mathrm{kg}\right)}{\mathrm{Cum}.\;\mathrm{Fluid}\;\mathrm{Intake}\;\left(g/\mathrm{kg}\right)}\times 100$$

where Cum. Fluid Intake is cumulative fluid intake during the rehydration period and Cum. Urine Output is cumulative urine output during the rehydration period.

To ensure a precise accounting of the water balance, cumulative evaporative water loss—encompassing sweating and insensible perspiration through the skin and respiratory tract—was calculated by subtracting the cumulative urine output from the total body weight loss incurred during the specific period.

Fluid intake and urine output were measured with an electronic balance; although a detailed analysis of individual drinking patterns or the exact timing of each drinking bout was not performed, cumulative fluid intake was determined at specific intervals (0.5 h, 1 h, 2 h, and 3 h after the onset of the rehydration period). Urine specific gravity was measured with a refractometer (Atago SUR-JE, Tokyo, Japan). Urine osmolality (U_osm_) was determined by freezing point depression (OSMOMAT 030, Gonotec; Berlin, Germany), and urine sodium (Na^+^), potassium (K^+^), and chloride (Cl^−^) ion concentrations were determined with an automated electrolyte analyzer with ion-selective electrodes (EA07, A&T, Fujisawa, Japan). Urinary Na^+^, K^+^, Cl^−^, and osmotically active substance excretion rates (U_Na_^+^$\dot{V}$, U_K_^+^$\dot{V}$, U_Cl_^−^$\dot{V}$, and U_osm_$\dot{V}$, respectively) were calculated by multiplying the urine flow rate by urine electrolyte concentrations or by urine osmolality. We determined urinary K^+^/Na^+^ as an index of the action of aldosterone [20]. We also calculated cumulative urine volume, as well as excretions of Na^+^, K^+^, Cl^−^ and osmotically active substances after fluid ingestion.

Tympanic temperature was measured using an infrared tympanic thermometer (Omron MC-510, Kyoto, Japan), with measurements performed at least twice. HR was measured continuously by photoplethysmography (Polar Verity Sense, Polar, Kempele, Finland). Data were sampled at the same time points as the other measurements (body temperature and subjective perception assessment) and used for subsequent analyses.

### 2.6. Subjective Perception

We performed subjective evaluations of thirst, mouth dryness, refreshment, sleepiness, thermal comfort, and stomach discomfort using VAS [15,21]. The line rating scale used to assess the subjective rating was 18 cm in length, with an intersecting line at 0 cm indicating “not at all” and another at 12 cm indicating “extremely so.” The subject was instructed to place a mark intersecting the analog scale at the point best representing their rating at the time. Subjects were instructed to mark on the line beyond 12 cm if they desired. We normalized the ratings so that 0 cm became 0% and 12 cm became 100%. The line to assess thermal comfort had two parallel lines, with an intersecting line on each at 0 cm indicating “neutral” and another at 12 cm indicating “extremely comfortable” and “extremely uncomfortable” on the other. Subjects were allowed to mark both lines, although this never happened. Thermal comfort ratings were normalized as above, with the exception that +100% indicated “extremely pleasant” and −100% indicated “extremely unpleasant”.

Following the initial bout of ingestion, subjects also rated the perceived intensity of sweetness, saltiness, bitterness, and sourness of the ingested solutions using a VAS.

### 2.7. Statistics

Values are presented as mean ± SEM. Data normality was assessed using the Shapiro–Wilk test. Continuous data were analyzed using a linear mixed model (LMM) with ingested fluid and time as fixed effects, and subject as a random intercept. LMM was employed to account for missing data. Although a random slope for time was initially considered, a random intercept-only model was preferred to maintain model parsimony and prevent convergence failures due to the sample size. To control the inflation of the Type I error rate across multiple repeated pairwise comparisons, LMM followed by Tukey’s post hoc test was applied to examine the main effects of trials and time points and their interaction on fluid balance data. Model assumptions and diagnostics for the LMMs, including the normality and homoscedasticity of residuals, were visually inspected and verified using Q-Q plots and residual plots. Preliminary analysis included sex as a fixed factor, but there was no significant main effect of sex or interactions related to gender (*p* > 0.05); thus, the results are presented as pooled data. For non-normally distributed ordinal data, a non-parametric repeated measures ANOVA (Friedman test) and subsequent Durbin–Conover pairwise comparisons were performed to determine differences in the perceived taste rating of the ingested fluid. A *p*-value of less than 0.05 indicated statistical significance. All analyses were performed using jamovi (version 2.7.24).

## 3. Results

### 3.1. Body Fluid Balance

**Figure 1:**
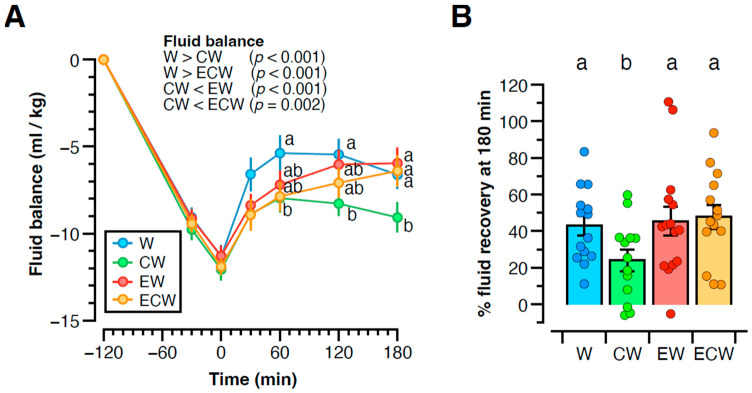
Total body fluid balance (**A**) during the dehydration and rehydration periods and percentage recovery of body fluid at 180 min after the onset of the rehydration period (**B**). Subjects were dehydrated by performing three bouts of stepping exercise for 20 min separated by 10 min of rest at 25 °C. Following the dehydration protocol, subjects ingested pure water (W), carbonated water (CW; gas volume, 3.43 ± 0.20 (mean ± SD)), electrolyte-added water (EW), or electrolyte-added CW (ECW; gas volume, 3.04 ± 0.29 (mean ± SD)) ad libitum for 180 min. EW and ECW contained 19 mEq/L Na^+^. Data are expressed as mean ± SEM. Significant differences among the ingested fluids—as analyzed by Tukey post hoc comparison after a linear mixed model (LMM), with ingested fluid and time as fixed effects and subject as a random intercept—are shown in the panels; labeled means without a common lowercase letter differ significantly (*p* < 0.05) among the experimental trials.

The total body fluid loss via evaporation and urine output during the dehydration protocol was 11.34 ± 0.65 g/kg body weight in the W trial, 12.07 ± 0.62 g/kg in the CW trial, 11.29 ± 0.57 g/kg in the EW trial, and 11.91 ± 0.47 g/kg in the ECW trial. The total fluid loss induced by the dehydration protocol was similar among the four experimental trials (Figure 1A). During the 180 min rehydration period, body fluid balance gradually increased in all groups (Figure 1A).

There were significant main effects of fluid type (*F*_3,247_ =19.78, *p* < 0.01) and time (*F*_4,246_ = 55.36, *p* < 0.01) on fluid balance, while no significant interaction between fluid and time was observed (*F*_12,246_ = 1.63, *p* = 0.084) (Figure 1A). Post hoc analysis revealed that the body fluid balance over the rehydration period in the W trial was significantly higher than in the CW and ECW trials (both *p* < 0.001) and in the EW (*p* < 0.001) and ECW (*p* = 0.002) trials than in the CW trial. In the W and CW trials, fluid balance plateaued after 60 min, and there was no subsequent increase. Notably, the net fluid balance remained lower in the CW than in the W trial. In contrast, the EW and ECW trials showed a slower initial increase but continued to increase, eventually reaching a level similar to the W trial at 180 min. Consequently, the body fluid balance at 180 min after the onset of the rehydration protocol was significantly more negative in the CW trial than in the W, EW and ECW trials (Figure 1A). The body fluid recovery ratio was 24.0 ± 6.0% in the CW, which showed a trend toward being lower compared to the W trial (42.9 ± 5.4%; mean difference: −20.66%, 95% CI: [−41.40, 0.08]%, *p* = 0.050), and was significantly less than in the EW (45.4 ± 8.0%; mean difference: −22.96%, 95% CI: [−43.24, −2.68]%, *p* = 0.021) and ECW (47.8 ± 6.6%; mean difference: −25.53%, 95% CI: [−46.27, −4.79]%, *p* = 0.010) trials (Figure 1B).

### 3.2. Cumulative Fluid Intake and Urinary Output During the 180 Min Rehydration Period

**Figure 2:**
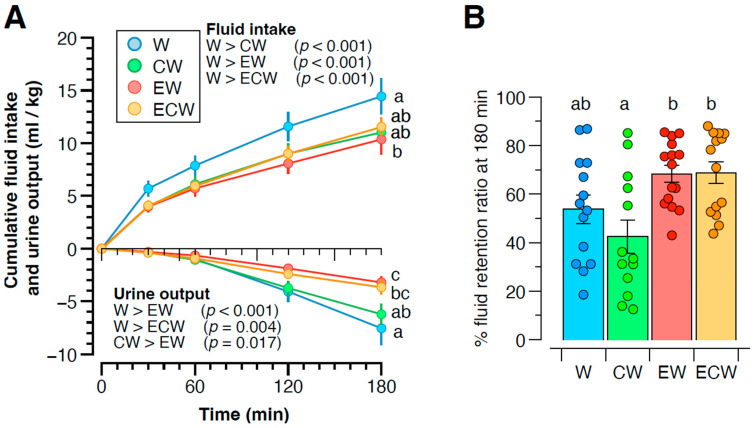
Cumulative fluid intake and urine output (**A**) and percent fluid retention ratio at 180 min after the onset of the rehydration period (**B**). Subjects were dehydrated by performing three bouts of stepping exercise for 20 min separated by 10 min of rest at 25 °C. Following the dehydration protocol, subjects ingested pure water (W), carbonated water (CW; gas volume, 3.43 ± 0.20 (mean ± SD)), electrolyte-added water (EW), or electrolyte-added CW (ECW; gas volume, 3.04 ± 0.29 (mean ± SD)) ad libitum for 180 min. EW and ECW contained 19 mEq/L Na^+^. Data are expressed as mean ± SEM. Significant differences among the ingested fluids—as analyzed by Tukey post hoc comparison after a linear mixed model (LMM), with ingested fluid and time as fixed effects and subject as a random intercept—are shown in the panels; labeled means without a common lowercase letter differ significantly (*p* < 0.05) among the experimental trials.

Fluid intake during the 180 min rehydration period after the dehydration protocol was 14.31 ± 1.61 g/kg body weight in the W trial, 11.02 ± 1.14 g/kg body weight in the CW trial, 10.36 ± 1.43 g/kg body weight in the EW trial, and 11.55 ± 0.89 g/kg body weight in the ECW trial (Figure 2A). There was a significant main effect of fluid type on cumulative fluid intake over the rehydration period (*p* < 0.001), while no significant interaction between fluid and time was observed (*p* = 0.526). Post hoc analysis revealed that the cumulative fluid intake over the rehydration period in the W trial was significantly higher than in the CW, EW, and ECW trials (all *p* < 0.001) (Figure 2A).

Furthermore, to explicitly evaluate the uncertainty and differences in cumulative fluid intake at the definitive endpoint, a pre-planned pairwise comparison was conducted at 180 min. This analysis demonstrated that although the overall main effect of fluid type was highly dominant across the entire session, the specific pairwise differences between the W trial and the carbonated/electrolyte trials at the final 180 min mark did not reach strict separate statistical significance (W vs. CW: mean difference: 3.68 g/kg, 95% CI: [−0.72, 8.07] g/kg, *p* = 0.128; W vs. EW: mean difference: 4.02 g/kg, 95% CI: [−0.18, 8.23] g/kg, *p* = 0.065; W vs. ECW: mean difference: 2.88 g/kg, 95% CI: [−1.38, 7.14] g/kg, *p* = 0.280). No meaningful differences in intake volume were observed among the CW, EW, and ECW trials at this final endpoint (all *p* > 0.50).

Cumulative urine output during the 180 min rehydration period was 7.56 ± 1.61 g/kg in the W trial, 6.22 ± 1.01 g/kg in the CW trial, 3.21 ± 0.58 g/kg in the EW trial, and 3.66 ± 0.68 g/kg in the ECW trial (Figure 2A). There was a significant main effect of ingested fluid type on cumulative urine output over the rehydration period (*p* < 0.001) and a significant interaction between ingested fluid and time (*p* = 0.002). Post hoc analysis revealed that the cumulative urine output over the rehydration period in the W trial was significantly greater than in the EW (*p* < 0.001) and ECW trials (*p* = 0.004) and also in the CW than in the EW trial (*p* = 0.017). At 180 min, cumulative urine output was greater in the W trial than in the EW and ECW trials (both *p* < 0.001), with no difference between W and CW trials. Additionally, at 180 min, cumulative urine output was greater in the CW trial than in the EW trial (*p* = 0.014), while no difference was found between the CW and ECW trials or between the EW and ECW trials (Figure 2A). To complement these final endpoints with structural uncertainty, a pre-planned pairwise comparison for cumulative urine output at 180 min confirmed that the urine output was significantly lower in the EW trial (mean difference from W: −4.31 g/kg; 95% CI: [−7.70, −0.92] g/kg, *p* = 0.008) and the ECW trial (mean difference from W: −3.89 g/kg; 95% CI: [−7.33, −0.46] g/kg, *p* = 0.021) compared to the W trial, with no significant difference observed between the W and CW trials (mean difference: 1.48 g/kg; 95% CI: [−2.06, 5.02] g/kg, *p* = 0.679). Furthermore, cumulative evaporative water loss during the 180 min rehydration period was 2.16 ± 0.21 gl/kg in the W trial, 1.80 ± 0.19 g/kg in the CW trial, 1.71 ± 0.26 g/kg in the EW trial, and 2.21 ± 0.22 g/kg in the ECW trial. There was no significant difference among the trials.

Taking the results of fluid intake and urine output, the net fluid retention ratio at 180 min was 53.7 ± 5.9% in the W trial, 42.5 ± 6.9% in the CW trial, 68.3 ± 3.5% in the EW trial, and 68.8 ± 4.5% in the ECW trial (Figure 2B). The fluid retention ratio in the CW trial was significantly lower than in the EW trial (mean difference: −25.24%, 95% CI: [−42.90, −7.59]%, *p* = 0.002) and the ECW trial (mean difference: −25.87%, 95% CI: [−43.89, −7.85]%, *p* = 0.002). No significant differences were observed between the W and CW trials (mean difference: 10.83%, 95% CI: [−7.19, 28.85]%, *p* = 0.381) or between the EW and ECW trials (mean difference: −0.63%, 95% CI: [−17.89, 16.64]%, *p* = 1.000) (Figure 2B). No significant difference was observed among the W, EW and ECW trials (Figure 2B).

### 3.3. Renal Responses

**Figure 3:**
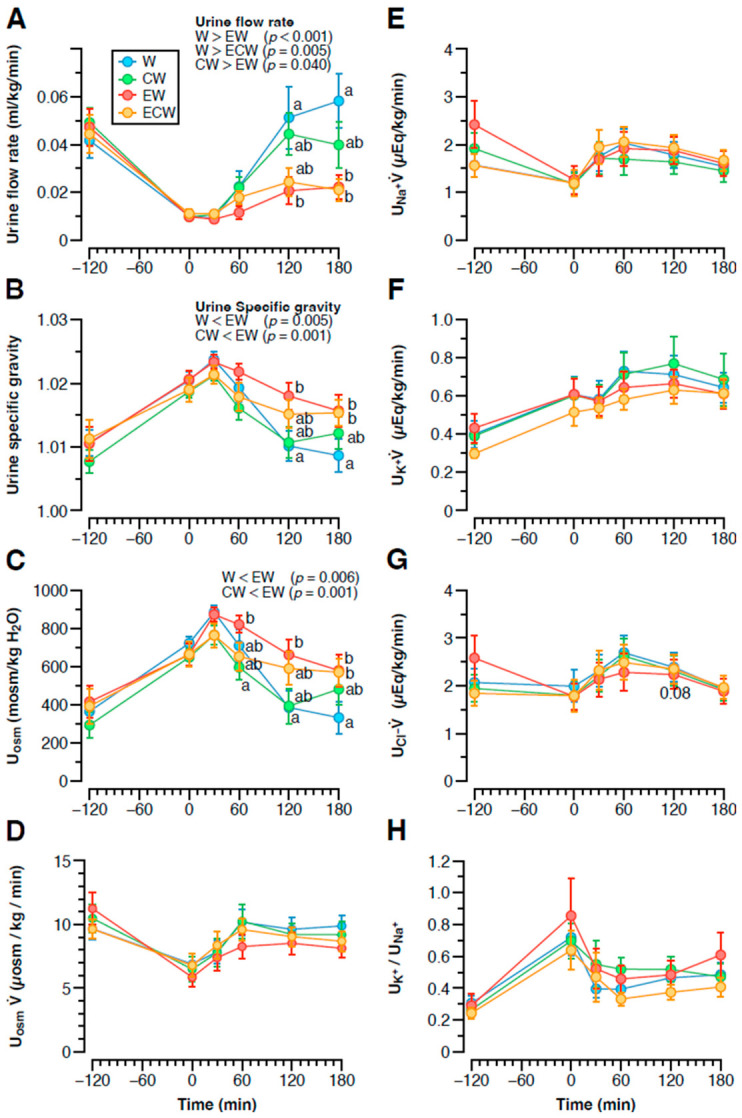
Urine flow rate (**A**), specific gravity (**B**), osmolality (U_osm_) (**C**), urinary osmotic substance excretion rate (U_osm_$\dot{V}$) (**D**), sodium excretion rate (U_Na_^+^$\dot{V}$) (**E**), potassium excretion rate (U_K_^+^$\dot{V}$) (**F**), chloride excretion rate (U_Cl_^−^$\dot{V}$) (**G**), and K^+^/Na^+^ ratio (**H**) during the dehydration and rehydration periods. Subjects were dehydrated by performing three bouts of stepping exercise for 20 min separated by 10 min of rest at 25 °C. Following the dehydration protocol, subjects ingested pure water (W), carbonated water (CW; gas volume, 3.43 ± 0.20 (mean ± SD)), electrolyte-added water (EW), or electrolyte-added CW (ECW; gas volume, 3.04 ± 0.29 (mean ± SD)) ad libitum for 180 min. EW and ECW contained 19 mEq/L Na^+^. Data are expressed as mean ± SEM. Significant differences among the ingested fluids—analyzed by Tukey post hoc comparison after a linear mixed model (LMM), with ingested fluid and time as fixed effects and subject as a random intercept—are shown in the panels; labeled means without a common lowercase letter differ significantly (*p* < 0.05) among the experimental trials.

The urine flow rate decreased from baseline during the dehydration period, and increased during the rehydration period (Figure 3A). Overall, there were significant main effects of ingested fluid type and time (both *p* < 0.001), and an interaction between ingested fluid type and time (*p* = 0.002), on urine flow rate. Post hoc analysis indicated that the urine flow rate was greater in the W trial than in the EW (*p* < 0.001) and ECW (*p* = 0.005) trials, and in the CW trial than in the EW trial (*p* = 0.004), over the 180 min rehydration period. The urine flow rate at 120 min after the onset of rehydration was greater in the W trial than in the EW trial (*p* = 0.016), while that at 180 min was greater in the W trial than in the EW and ECW trials (both *p* < 0.001) (Figure 3A).

Urine specific gravity (Figure 3B) and osmolality (Figure 3C) increased during the dehydration protocol, and decreased during the rehydration period. There were significant main effects of ingested fluid type on urine specific gravity and osmolality (both *p* < 0.001) and time on urine specific gravity and osmolality (both *p* < 0.001), while a significant interaction between these variables was observed on urine osmolality (*p* = 0.029) but not on urine specific gravity. Post hoc analysis revealed that urine specific gravity and osmolality were significantly lower in the W trial than in the EW trial (*p* = 0.005 for specific gravity and 0.006 for osmolality), as well as in the CW trial than in the EW trial (both *p* = 0.001) (Figure 3B,C).

Urine specific gravity was higher in the EW trial than in the W trial (*p* = 0.016) at 120 min after the onset of the rehydration period and higher in the EW (*p* = 0.028) and ECW (*p* = 0.043) trials than in the W trial at 180 min (Figure 3B). Urine osmolality was higher in the EW trial than in the CW trial at 60 min after the onset of the rehydration period (*p* = 0.047), higher in the EW trial than in the W trial at 120 min (*p* = 0.037), and higher in the EW (*p* = 0.023) and ECW (*p* = 0.036) trials than in the W trial at 180 min (Figure 3C).

**Figure 4:**
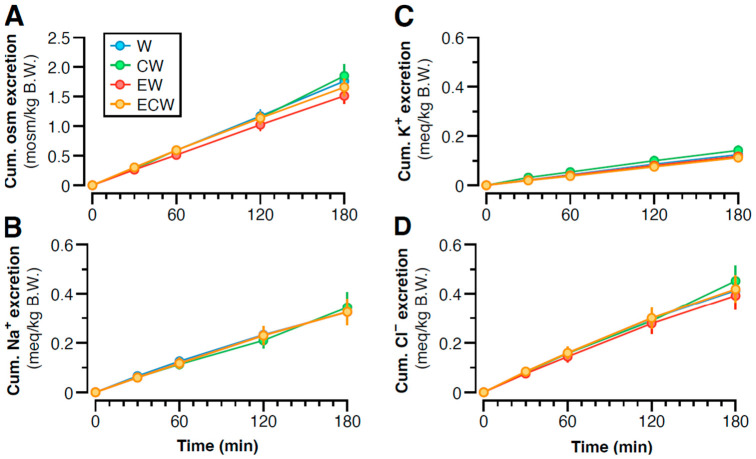
Cumulative (Cum.) osmotic substance (Osm) (**A**), Na^+^ (**B**), K^+^ (**C**), and Cl^−^ (**D**) excretions during the rehydration period. Subjects were dehydrated by performing three bouts of stepping exercise for 20 min separated by 10 min of rest at 25 °C. Following the dehydration protocol, subjects ingested pure water (W), carbonated water (CW; gas volume, 3.43 ± 0.20 (mean ± SD)), electrolyte-added water (EW), or electrolyte-added CW (ECW; gas volume, 3.04 ± 0.29 (mean ± SD)) ad libitum for 180 min. EW and ECW contained 19 mEq/L Na^+^. Data are expressed as mean ± SEM.

There were no differences in U_osm_$\dot{V}$ (Figure 3D), U_Na_^+^$\dot{V}$ (Figure 3E), U_K_^+^$\dot{V}$ (Figure 3F), U_Cl_^−^$\dot{V}$ (Figure 3G), or U_K_^+^/U_Na_^+^ (Figure 3H) among the trials, nor in cumulative osmotic substance (Figure 4A), sodium (Figure 4B), potassium (Figure 4C), or chloride (Figure 4D) excretions during the rehydration period among the trials.

### 3.4. Physiological and Psychological Responses

**Figure 5:**
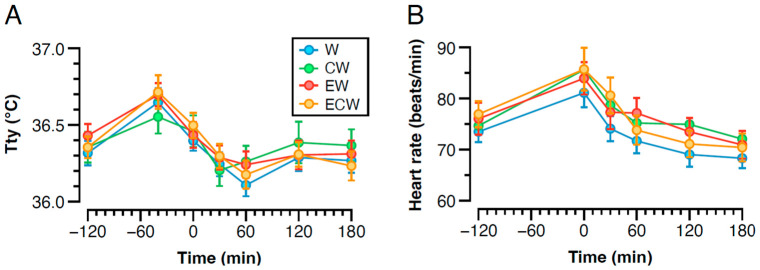
Tympanic temperature (Tty) (**A**) and heart rate (**B**) during the dehydration and rehydration periods. Subjects were dehydrated by performing three bouts of stepping exercise for 20 min separated by 10 min of rest at 25 °C. Following the dehydration protocol, subjects ingested pure water (W), carbonated water (CW; gas volume, 3.43 ± 0.20 (mean ± SD)), electrolyte-added water (EW), or electrolyte-added CW (ECW; gas volume, 3.04 ± 0.29 (mean ± SD)) ad libitum for 180 min. EW and ECW contained 19 mEq/L Na^+^. Data are expressed as mean ± SEM.

Throughout the experiment, tympanic temperature (Figure 5A) and HR (Figure 5B) were not different among the four trials.

**Figure 6:**
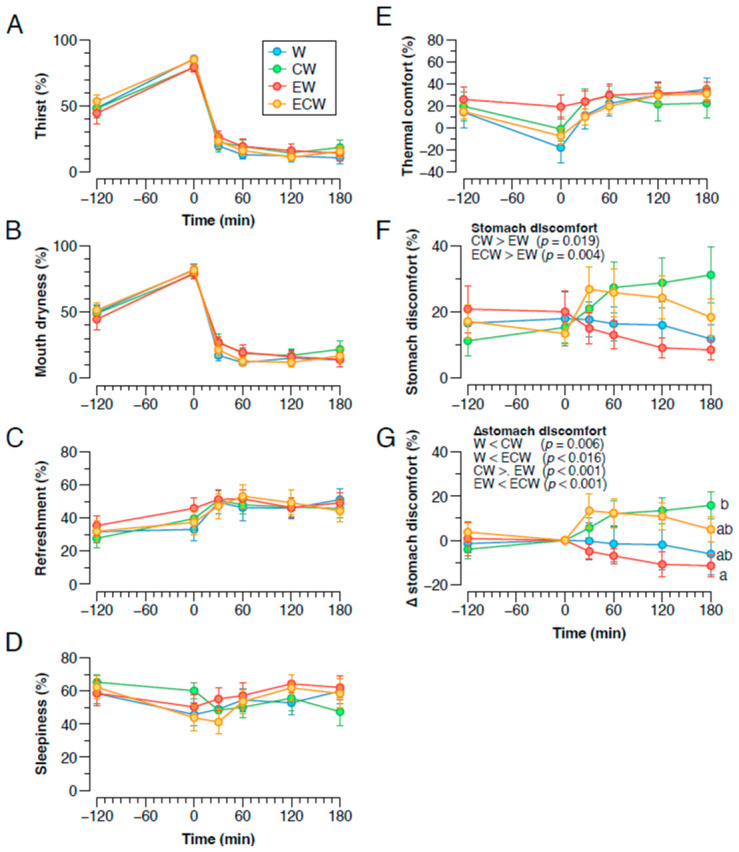
Subjective rating of perceived thirst (**A**), mouth dryness (**B**), refreshment (**C**), sleepiness (**D**), thermal comfort (**E**), stomach discomfort (**F**), and the change in stomach discomfort from pre-rehydration levels (∆stomach discomfort) (**G**) during the dehydration and rehydration periods. Subjects were dehydrated by performing three bouts of stepping exercise for 20 min separated by 10 min of rest at 25 °C. Following the dehydration protocol, subjects ingested pure water (W), carbonated water (CW; gas volume, 3.43 ± 0.20 (mean ± SD)), electrolyte-added water (EW), or electrolyte-added CW (ECW; gas volume, 3.04 ± 0.29 (mean ± SD)) ad libitum for 180 min. EW and ECW contained 19 mEq/L Na^+^. Data are expressed as mean ± SEM. Significant differences among the ingested fluids—analyzed by Tukey post hoc comparison after a linear mixed model (LMM), with ingested fluid and time as fixed effects and subject as a random intercept—are shown in the panels; labeled means without a common lowercase letter differ significantly (*p* < 0.05) among the experimental trials.

Subjective thirst (Figure 6A), mouth dryness (Figure 6B), refreshment (Figure 6C), sleepiness (Figure 6D), and thermal comfort (Figure 6E) were not different among the four trials. In contrast, stomach discomfort during the rehydration period was significantly affected by ingested fluid (Figure 6F). Overall, there was a significant main effect of ingested fluid (*p* = 0.002) and a significant interaction between ingested fluid and time (*p* = 0.031) on stomach discomfort. Post hoc analysis indicated that stomach discomfort was higher in the CW (*p* = 0.019) and ECW (*p* = 0.004) trials than in the EW trial. Furthermore, the change in stomach discomfort (∆stomach discomfort) from pre-rehydration levels was significantly affected by the ingested fluid type (*p* < 0.001), with no interaction between ingested fluid and time (Figure 6G). Post hoc comparison revealed that ∆stomach discomfort was greater in the CW (*p* = 0.006) and ECW (*p* = 0.016) trials than in the W trial and in the CW (*p* < 0.001) and ECW (*p* < 0.001) trials than in the EW trial. More specifically, ∆stomach discomfort at 180 min after the onset of the rehydration period was significantly higher in the CW trial than in the EW trial (*p* = 0.044).

**Figure 7:**
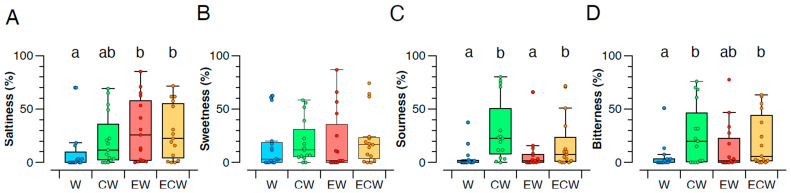
Perceived taste ratings for each rehydration fluid after the first bout of ingestion following the dehydration period. Ratings include saltiness (**A**), sweetness (**B**), sourness (**C**), and bitterness (**D**). W, pure water; CW, carbonated water; EW, electrolyte-added water; and ECW, electrolyte-added carbonated water. Data are expressed as mean ± SEM. Labeled means without a common letter differ significantly (*p* < 0.05) among the experimental trials, as analyzed by a non-parametric repeated measures ANOVA (Friedman test) followed by Durbin–Conover pairwise comparisons.

Regarding the perceived taste of each solution, as rated by subjects immediately after the first bout of ingestion, saltiness ratings were significantly higher for EW (*p* = 0.048) and ECW (*p* = 0.019) than for W (Figure 7A). Sweetness ratings were not different among the four fluids (Figure 7B). Sourness ratings were significantly higher for CW (*p* < 0.001) and ECW (*p* = 0.03) than for W; additionally, they were higher for CW (*p* = 0.007) and ECW (*p* = 0.038) than for EW (Figure 7C). Ratings for bitterness were greater for CW (*p* = 0.006) and ECW (*p* = 0.008) than for W (Figure 7D).

## 4. Discussion

The primary objective of this study was to investigate the effects of carbonation and electrolyte supplementation on rehydration efficiency during ad libitum fluid intake following mild hypohydration. We found that the ad libitum ingestion of carbonated water (CW) significantly reduced the fluid recovery ratio—and thus that it was less effective for rehydration—compared to plain water (W) and electrolyte-added water (EW). In contrast, the fluid recovery ratio after ad libitum ingestion of electrolyte-added carbonated water (ECW) did not differ from that of W or EW (Figure 1A,B). These results indicate that although ad libitum rehydration with plain carbonated water is less effective, electrolyte supplementation improves the restoration of body fluid balance following mild thermal dehydration.

The reduced fluid intake during the rehydration period in the CW trial, compared to the W trial, is the primary factor that contributed to the lower fluid recovery, whereas cumulative urinary output in the CW trial did not differ from that in the W trial. In contrast, when comparing the CW trial to the EW and ECW trials, the lower fluid recovery in the CW trial is primarily attributed to lower fluid retention, given that cumulative fluid intake was comparable among these carbonated and/or electrolyte-added trials (Figure 2A,B).

Osmoregulation takes priority over volume regulation in maintaining body fluid homeostasis [15,22,23]. Thus, after adjusting plasma osmolality to pre-hypohydration levels by ingesting water without electrolyte supplementation, any further ingested fluid dilutes the body fluids and enhances diuresis, resulting in the excretion of a volume of water comparable to that ingested [15]. Accordingly, electrolyte supplementation—especially with sodium—is indispensable for achieving full rehydration following thermally induced hypohydration, which involves the loss of both water and salts. In the present study, fluid balance in the W and CW trials plateaued after 60 min (Figure 1A), indicating that fluid intake and urine output were in equilibrium and suggesting that dilution-induced diuresis occurred after this time point in these trials. Although we did not measure plasma osmolality in the present study, this plausible interpretation is strongly supported by the aforementioned results, as well as the changes in urine specific gravity and urine osmolality, which were lower in the W and CW trials than in the EW trial (Figure 3B,C). Importantly, the urinary electrolyte excretions and K^+^/Na^+^ ratio were not different among the trials. This absence of differences in electrolyte excretion strongly suggests that the observed differences in urine flow and osmolality were not driven by changes in renal solute handling. Instead, these findings support the interpretation that the diuresis was primarily regulated by the osmoregulatory system, rather than the volume regulatory system via the renin–angiotensin–aldosterone system. However, equilibrated fluid balance was more negative in the CW than in the W trial (Figure 1B). The mechanisms underlying the differences in equilibrated fluid balance between the CW and W trials would remain unexplained if fluid balance were solely determined by osmoregulation. These mechanisms could be further elucidated by measuring plasma osmolality and arginine vasopressin levels. In contrast to the ingestion of W and CW, fluid balance continued to recover over the 180 min rehydration period through the ingestion of EW and ECW. These data further support the importance of electrolytes—especially sodium—for full recovery when rehydrating with carbonated water.

Regarding the final fluid retention ratio, those of EW and ECW appeared numerically higher than that of W, although the difference did not reach statistical significance. From a physiological perspective, this numerical elevation highlights the potent impact of electrolyte (sodium) supplementation in promoting renal water retention, which effectively compensates for the carbonation-induced reduction in voluntary fluid intake. This suggests that adding electrolytes to carbonated water optimizes the efficiency of rehydration per unit volume ingested, allowing individuals to achieve a comparable level of overall body fluid recovery despite consuming less fluid.

Fluid intake is regulated by body fluid status, fluid palatability, and information from the oral and gastrointestinal tract [24,25]. In the present study, thirst ratings were not different among the trials (Figure 6A). Interestingly, fluid intake was significantly lower in the CW, EW, and ECW trials than in the W trial, with no additive effect of carbonation and electrolyte provision on intake in the ECW trial. We did not directly assess fluid palatability in the present study, but we assessed perceived taste just after the first bout of ingestion following the dehydration protocol. The different perceived tastes, especially saltiness, sourness, and bitterness, might contribute to the lower fluid intake in the CW, EW, and ECW trials than in the W trial during the rehydration period (Figure 7). Importantly, saltiness, sourness, and bitterness ratings were not different between CW and ECW. Additionally, the stomach discomfort ratings might contribute to the reduced fluid intake in the CW and ECW trials compared to the W and EW trials [24,26] (Figure 6F,G). These psychological factors are likely to contribute to the different fluid intake by the fluid type.

Compared to plain water ingestion, carbonated water ingestion (150 mL for men and 100 mL for women) in resting humans exposed to heat stress [11] and post-exercise in heat [12] reportedly increased mean arterial pressure, cerebral blood flow, and oral and whole-body exhilaration and decreased sleepiness. In the present study, we failed to find any difference in refreshment or sleepiness among the trials. The different results are attributed to the differences in experimental protocols. Those studies examined the effect of a bolus ingestion of carbonated water and short-term responses thereafter, while our study did not examine these responses.

Thermoregulatory responses to heat stress are reportedly inhibited by hypohydration, which consequently increases the risk of heat-related illness [3,4,5,6]. In the present study, however, we failed to find any significant differences in tympanic temperature, thermal sensation, or heart rate among the trials, even though body fluid balance recovery was less efficient in the CW trial compared to the W, EW, and ECW trials. The most likely reason for this lack of differentiation is that the level of hypohydration in our subjects was too mild to trigger measurable thermoregulatory impairment. More specifically, the hypohydration level was approximately 10 g/kg body weight, or 1% of total body weight—a degree of mild hypohydration that might typically occur during summer without strenuous physical activity [27]. Given that a deficit of 2% of body mass is generally recognized as the physiological threshold for impaired thermoregulation [5,6], our results align with this established benchmark. Nonetheless, it remains possible that the cumulative effects of hypohydration over a more prolonged duration could eventually have a detrimental impact on thermoregulatory function.

### Limitations

The present study is a practical, application-oriented study designed to clarify the effects of ad libitum intake of carbonated and electrolyte-supplemented water on recovery from mild hypohydration. As the approach is primarily phenomenological, the underlying physiological mechanisms have not been fully investigated. Specifically, a primary limitation is the lack of direct measurements for plasma osmolality, plasma volume, fluid- and thirst-regulating hormones (e.g., arginine vasopressin and aldosterone), and direct sweat sodium loss during the dehydration protocol. Furthermore, our experimental design utilized only one specific sodium concentration (19 mEq/L) and one carbonation level (approx. 3.0–3.4 gas volume).

Another limitation regarding external validity is that the subjects in the present study were all young women and men; thus, we are not able to conclude that the results are applicable to children or older and middle-aged people. The risk for heat-related illness is reportedly greater in older adults compared to younger people [28], with older adults showing less effective rehydration following thermally induced hypohydration [29].

To establish broader mechanistic confirmation and external validity, future research directions should expand on these boundaries. It is necessary to investigate different populations, including older adults who require more effective fluid restoration, as well as varying severities of dehydration (beyond the mild ~1% level tested here). Additionally, future studies should examine different fluid ingestion protocols (e.g., prescribed or bolus drinking versus ad libitum intake) and a wider range of sodium and carbonation concentrations to determine optimal rehydration formulations.

Finally, we speculate that the perceived taste of ingested fluid may be associated with the ingestive behavior and the fluid volume ingested during rehydration. In the present study, to minimize interference with voluntary consumption, sensory evaluation was conducted only once, immediately after the initial bout. Examining the correlation between sensory attributes and intake patterns will elucidate the characteristics of CW ingestive behavior.

## 5. Conclusions

While carbonated water is often preferred for its palatability and refreshing qualities, relying solely on plain carbonated water for post-dehydration recovery may lead to insufficient fluid replacement. However, combining carbonation with electrolytes (ECW) represents an effective strategy for improving fluid restoration by enhancing fluid retention. It allows individuals to enjoy the sensory benefits of carbonation while achieving efficient fluid restoration through enhanced renal retention, even with a lower total intake volume. This makes electrolyte-added carbonated water a potentially useful rehydration beverage within the scope of mild hypohydration in young healthy adults.

## Figures and Tables

**Table 1:** Anthropometric variables of subjects.

	Female (*n* = 8)	Male (*n* = 7)	Mean (*n* = 15)
Age	22.9 ± 0.6	22.3 ± 0.6	22.6 ± 0.4
Height (cm)	158.6 ± 1.9	166.7 ± 2.7	162.6 ± 2.0
Body weight (kg)	49.9 ± 0.9	61.1 ± 3.2	55.2 ± 2.1
BMI	19.9 ± 0.5	21.9 ± 0.6	20.8 ± 0.4

Data are shown as mean ± SEM.

**Table 2:** Composition of rehydration fluids (mg/100 mL).

Ingested Fluid	Na^+^	K^+^	Ca^2+^	Mg^2+^	Cl^−^	Silica	Gas Volume	pH
**W**	0	0	0	0	0	0	0	5.51
**CW**	0	0	0	0	0	0	3.43 ± 0.20 (mean ± SD)	3.72
**EW**	44 19 mEq/L	17 4.3 mEq/L	2.5 1.2 mEq/L	1.1 0.9 mEq/L	19 5.4 mEq/L	5.1	0	7.24
**ECW**	43 19 mEq/L	17 4.3 mEq/L	2.5 1.2 mEq/L	1.1 0.9 mEq/L	20 5.6 mEq/L	5.3	3.04 ± 0.29 (mean ± SD)	5.51

W: pure water, CW: carbonated water, EW: electrolyte-added water, ECW: electrolyte-added carbonated water. EW and ECW contained other anions such as citrate and bicarbonate.

## Data Availability

Data will be made available from the corresponding author upon reasonable request.
